# Books Reviewed

**Published:** 1865-11

**Authors:** 


### Books Reviewed.

LECTURES ON INFLAMMATION: Being the first Course delivered before the College of Physicians of Philadelphia, under the Bequest of Dr. MUTTER. By JOHN H. PACKARD, M. D., Author of "A Manual of Minor Surgery," Translator of Malgaigne's "Traite des Fractures," Secretary of the College of Physicians, etc., etc. Philadelphia: J. B. LIPPINCOTT & Co. 1865.

The author of these Lectures was appointed by the College of Physicians of Philadelphia to deliver the first three courses of lectures under the bequest of Dr. Mütter, the distinguished surgeon of Philadelphia, and Professor in the College of Physicians, who bequeathed to it his pathological museum, together with a fund for its preservation, and for the endowment of a lectureship. In ten lectures, the author has considered Inflammation, in all its varied forms and relations, giving the most recent views of modern pathology. The subjects of cell growth, animal heat, nutrition, reflex nervous influence, and various other similar topics are discussed with great clearness and ability. Inflammation, its terminations, the nature and sources of its products, its causes, its purposes and the various theories concerning it, are all presented in a brief but comprehensive manner. All the various phenomena of inflammation are considered with most commendable exactness.

The tenth lecture is upon the therapeutics of inflammation—general relations of Pathology and Therapeutics, and the effects of cold, warmth and moisture, counter irritation, derivatives, bleeding, low diet, anodynes, counter irritants, and a great many other topics, which we have not space to enumerate.

These lectures are written in direct and forcible style, and embody the most recent pathological views upon the various questions discussed. There are so many topics embraced in inflammation and its terminations and associations, that we almost propose a new name for the work; the Philosophy of Life would scarcely admit of a wider range, since all the vexed questions in pathology are in some way connected with the phenomena of inflammation. The work is worthy a careful perusal, and should be in the hands not only of students of medicine, but of physicians, who cannot fail to obtain from it valuable practical suggestions.

---

Annual Address before the Medical Society of the County of Oneida. By C. B. Coventry, M. D., President of the Society. Published by the Society.

This Address is upon Tubercular Phthisis, which he says "consists in the deposition in the tissues of organs and on the surface of membranes of small bodies, first appearing simply as a thickening and opacity of the part, then of small bodies termed miliary tubercle, which may remain stationary or unaltered, or may gradually increase in size, become yellowish or cheesy, and finally soften and be gradually absorbed, or more frequently inflammation and ulceration occur, when they may be discharged.

Origin.—Pathologists differ in opinion, whether these bodies consist of original nutritive matter which is imperfectly organized, or of effete matter which the system is incompetent to eliminate, or of actual transformation of tissue; it is however, admitted that in either case it is an evidence of want of vitality or vigor in the system.

1st.—Tuberculosis is a constitutional, and not a local affection. Tubercles are found in almost every tissue and organ, as the membranes of the brain, the lungs, liver, peritoneum, intestines, etc.

2d.—Tuberculosis is hereditary. Dr. Armstrong in his Lectures says he has found tubercles in children at the breast, who had died suddenly of acute disease. No doubt exists that a peculiarity of constitution which predisposes the offspring to the disease, is transmitted from parent to child.

3d.—Tuberculosis may be produced, *de novo*, where there is no hereditary predisposition.

4th.—Tubercles may go on increasing and multiplying, or their increase may be arrested, and they remain an indefinite period of time, with no further disturbance of health than what arises from mechanical obstruction; this obstruction is of itself seldom a cause of death. I have met with one case of the kind; in this instance both lungs seemed masses of miliary tubercles, but there was no appearance of inflammation, or indications of softening.

The following changes it is supposed may take place in tubercles, when their progress is arrested and inflammation prevented:—1. Liquefaction; 2. Absorption; 3d. Fatty degeneracy, granular degeneracy, calcarious degeneracy, shriviling, pigmentum degeneracy, sequestration. We have no positive proof of the absorption of nascent tubercles. When inflammation supervenes in the lungs of a patient having tuberculosis, and terminates in ulceration, it constitutes Tubercular Phthisis; it should, however, be remembered that Pneumonia occurring in tubercular lungs, is usually more limited in extent, being usually or frequently confined to one lung, or a single lobe of the lung.

The remote or predisposing cause of tubercular phthisis, is a tubercular condition of the system.

The causes of tuberculosis, are:

1—Hereditary predisposition.

2—Insufficient or unwholesome food.

3—Insufficient clothing.

4—A variable and damp climate.

5—Confinement in dark, ill-ventilated and crowded apartments.

6—Irregular habits.

7—Exhausting drains, exhausting discharges, too long nursing.

8—Certain occupations, as stone cutting, etc.

9—Exhausting mental application and anxiety of mind.

The immediate or exciting causes of tubercular phthisis, are all those causes which tend to produce pneumonia under other circumstances."

He says, under the head of Prevention, "Mothers who are consumptive should not nurse their children, but a healthy wet nurse should be substituted for the mother; if this cannot be done, the child should be weaned as early as the seventh or eighth month, and nourished on healthy cows' milk. The child should be carefully protected from the inclemency of the weather, but should not be confined to the house; on the contrary, they should be permitted to spend as much time as convenient in the open air, should retire early to bed, and be up in season in the morning. The food should be light and nutritious, but not stimulating. In such children more care is necessary to prevent, than to stimulate or excite mental application; they should not be sent early to school, and the parents should see that the mind is not over-taxed. Girls who are delicate, if constitutionally inclined to consumption, should be kept from school from the age of twelve to fifteen, the preservation of health at this period is the most important consideration, and all the energies of the system are required to sustain the rapid growth and development which usually takes place at this age. Nutritious food should be used, warm clothing, plenty of exercise in the open air, cheerful company, and freedom from care and anxiety of mind are important. Young persons who are predisposed to phthisis, should be careful in selecting a profession or occupation that would not confine them too much, or be attended with much anxiety of mind. Formerly it was customary, if a boy was feeble or delicate, to give him a profession, or put him in a store, or to some sedentary mechanical occupation, on the ground that he was not strong enough for a farmer. Directly the reverse should have been adopted; if a boy is feeble or delicate, an occupation should be selected which would tend to improve his physical health and strength. Many young men have no doubt fell victims to this mistaken idea. When the predisposition is strong, or a person is actually threatened with the disease, a change of customary occupation, and even a change of climate may be useful.

Traveling, when the person can indulge in it, riding on horseback, systematic exercise of any kind in the open air, carried to the extent of moderate fatigue, is useful. If the appetite is good, the bowels regular, and the other functions of the system properly performed, the patient needs no medicine. The food should be nutritious, but not heating or stimulating; ardent spirits of every kind should be avoided. They should be warmly clothed to protect them against the sudden changes of the weather, and should be particularly careful to guard against all those causes which usually produce influenza, or what is usually termed taking cold; if, notwithstanding their care, they should contract a cold, they should at once apply for relief, and not permit it to run on without treatment,

It is a mooted question whether consumption is contagious. With the ancients there was no doubt on the subject, and even now in the southern part of Europe, the belief in its contagious nature is so strong that they burn the bed and bedding of consumptive patients. Modern writers have mostly denied its contagious nature ; but Dr. Armstrong says that he is satisfied, that either from the irritating effects of the effluvia, or its poisonous character, it tends to produce the disease. Consumption is certainly not contagious in the same sense as small-pox, measles, etc., but that it may be communicated or produced by intimate intercourse, I have no doubt. We all know how common it is for whole families to be carried off, one after another, by this disease; it may be said that they were all probably suffering from the same predisposition and tendency to disease, and that the fatigue of nursing, broken sleep, and anxiety of mind, were sufficient to account for the circumstance, without the aid of contagion. When a pupil, I was cautioned by my preceptor, against making post mortem examinations of consumptive patients, for fear of contracting the disease. The late Professor Willoughby told me that his brother, who was a stout healthy man, caught the disease by nursing a sick daughter. I have seen so many cases where husband or wife have evidently contracted the disease, the one from the other, where there was no previous predisposition, that I have no doubt of its being communicated. Where there is a predisposition to the disease, the immediate connections should never nurse a consumptive patient.

The anxiety of mind, the disturbed rest, and confinement, would all tend to produce the disease, aside from contagion. In no case should they be permitted to sleep in the same bed, and care should be taken that the apartment is well ventilated. Whatever breaks up the general strength and health, tends to develop tubercles, if they previously existed, or to produce them, *de novo*. Dr. Armstrong says: "If you maintain the general strength in the children of families where consumption prevails, and also in adults, you will prevent the occurrence of the disease; break up the general strength, and the disease will be developed." This shows how cautious we should be in the use of active medicines in consnmptive families, when suffering from other diseases, unless it be one of vital importance.

We also make a brief quotation of "treatment":

As has been seen, we have in tubercular phthisis three distinct stages, viz: 1st. Tuberculosis simply, without inflammation; 2d. Tuberculosis complicated, with inflammation; 3d. Tuberculosis complicated, with suppuration and ulceration of the lungs. It is not often the physician is consulted in the first stage. The indications are to arrest the farther progress of the disease, and particularly to guard against the occurrence of inflammation. This is to be effected by the use of all those means recommended for the prevention of the disease, viz: change of occupation, change of climate, horse exercise, a nutricious diet, warm clothing, regular habits, and living as much as possible in the open air. If the appetite is good, the bowels regular, and the patient rests well, medicine is not needed, and would be more likely to do harm than good. If the apppetite is defective, and the patient becomes emaciated, tonics and stimulants may be necessary. The cod-liver oil, when it can be taken, is well adapted to nourish the system; when this can not be taken, cream is now used as a substitute. Ale or porter, or wine, and even alcoholic liquors, as brandy or Bourbon whisky, may be advisable. The indiscriminate manner in which whisky is now used by patients, and even recommended by some physicians without reference to the actual condition, is much to be regretted. Alcoholic drinks are no doubt useful in some cases, but I fear their general use at the present time in consumption, is doing more harm than good. I am inclined to think that the sun's rays have more influence on health than we are accustomed to imagine. The influence of the sunlight on vegetables is well known ; it is also known that tubercles may be produced in cows in dark and ill-ventillated stables, with meager and insufficient nourishment, and that in our large cities it is found very common among children living in dark, damp and imperfectly ventilated cellars, and often with insufficient food. Almost every person has experienced a difference in their feeling, between a dark, cloudy day, and one of bright sunshine. In our damp climate, we need sunshine more than shade. I am satisfied that the practice of surrounding our houses with shade trees so as to exclude the sun and air, is prejudicial to health, as also the pernicious custom of shutting out the sun from apartments, for fear of fading the carpets. If the ladies think more of the colors of the carpet than of the daughters' cheeks, they should do as a distinguished judge of our own State is said to have done in early life, viz: to keep the carpet rolled up, and only put it down on state occasions. Moderate labor, carried to the extent of slight fatigue, is no objection in this stage of the disease."

This address is so practical, so eminently scientific and sensible, that we should be glad to copy it entire. We have indulged this wish quite largely, and the main teachings of the paper are before our readers. It is the ripe fruit of an eminently practical mind, and Dr. Coventry has conferred a real benefit, by his plain, common sense teaching of the nature, causes, prevention and treatment of Tubercular Phthisis.

